# Prevalence and Risk Factors of Incidental Findings in Brain MRIs of Healthy Neonates—The FinnBrain Birth Cohort Study

**DOI:** 10.3389/fneur.2019.01347

**Published:** 2020-01-08

**Authors:** Venla Kumpulainen, Satu J. Lehtola, Jetro J. Tuulari, Eero Silver, Anni Copeland, Riikka Korja, Hasse Karlsson, Linnea Karlsson, Harri Merisaari, Riitta Parkkola, Jani Saunavaara, Tuire Lähdesmäki, Noora M. Scheinin

**Affiliations:** ^1^FinnBrain Birth Cohort Study, Turku Brain and Mind Center, Institute of Clinical Medicine, University of Turku, Turku, Finland; ^2^Department of Psychiatry, Turku University Hospital, University of Turku, Turku, Finland; ^3^Department of Psychology, University of Turku, Turku, Finland; ^4^Centre for Population Health Research, Turku University Hospital, University of Turku, Turku, Finland; ^5^Department of Child Psychiatry, Turku University Hospital, University of Turku, Turku, Finland; ^6^Department of Future Technologies, University of Turku, Turku, Finland; ^7^Center of Computational Imaging and Personalized Diagnostics, Case Western Reserve University, Cleveland, OH, United States; ^8^Department of Radiology, Turku University Hospital, University of Turku, Turku, Finland; ^9^Department of Medical Physics, Turku University Hospital, Turku, Finland; ^10^Department of Pediatric Neurology, Turku University Hospital, University of Turku, Turku, Finland

**Keywords:** infant, incidental finding, MRI, subdural hemorrhage, delivery method

## Abstract

**Background:** Birth is a traumatic event with molding forces directed to the fetal skull, which may result in intracranial hemorrhages. However, the knowledge on prevalence and risk factors of incidental brain magnetic resonance imaging (MRI) findings in infants is still inconclusive.

**Methods:** The prevalence and nature of incidental MRI findings were assessed in a birth cohort of 175 asymptomatic infants. The role of delivery method as well as other potential risk factors for intracranial hemorrhages were evaluated. The infants underwent 3T MRI at the age of 2–5 weeks, and the neurological status of the infants with an incidental finding was evaluated by a pediatric neurologist. Information on the delivery method, duration of delivery, parity, used anesthesia, oxytocin induction, and Apgar score was gathered to evaluate their association with the prevalence of hemorrhages.

**Results:** Incidental intracranial hemorrhages were detected in 12 infants (6.9%), all following spontaneous or assisted vaginal delivery. Vacuum-assistance was found to be a risk factor for subdural hemorrhages with an odds ratio (OR) of 4.7 (95% CI [1.18; 18.9], *p* = 0.032). All infants were evaluated to develop normally by their clinical status.

**Conclusions:** Incidental intracranial hemorrhages are relatively common among infants born by vaginal delivery. They are often of little clinical significance within the first years of life and have unlikely consequences for later neurodevelopment either. Despite their benign character, investigators should be prepared to share this information with parents competently as the findings can cause parental anxiety, and especially as the popularity of MRI as a research tool is increasing.

## Introduction

Incidental intracranial hemorrhages are common findings in neonatal magnetic resonance imaging (MRI). This is not surprising given the remarkable molding and deformation of the fetal head in the birth canal (1, 2). Despite being common, the relevance of birth-related intracranial hemorrhages is still partially unclear, as are also risk factors behind them.

The estimated prevalence of intracranial hemorrhages in term asymptomatic healthy newborn populations deviate greatly from 8.1 to 46% in previous studies (3–5). Subdural hemorrhages are most common, and it is customary to divide them into two groups according to their location—supratentorial and infratentorial. While in most studies infratentorial hemorrhages form the majority (4–6), one study reported a high prevalence of supratentorial hemorrhages (3). Some studies have observed exclusively subdural hemorrhages (3, 5), while other more infrequently reported findings include subarachnoidal (4) and intraparenchymal hemorrhages (4, 6). Apart from hemorrhages, cysts (e.g., pineal and caudothalamic cysts) are commonly (1.9–57%) reported benign incidental intracranial findings (7, 8).

Neonatal intracranial hemorrhages may appear in multiple locations (3, 4). Subdural hemorrhages, detected immediately after birth, have been described most often to situate posteriorly (3–5, 9). This location differs from that of subdural hemorrhages related with non-accidental head injuries (“shaken baby syndrome”), that typically present in the interhemispheric fissure and over the hemispheres (10, 11). Birth-related intracranial subdural hemorrhages are often widely distributed (forming a thin film) and seldom have indications for clinical interventions (12, 13). However, it remains elusive whether e.g., location of the hemorrhage could play a role in possible consequences.

Vaginal delivery is a risk factor for intracranial hemorrhages (3–6), while the effects of assisted delivery and other obstetric factors remain less clear. Some studies indicate vacuum extraction as a risk factor (14, 15), while others have not detected an association (3, 4, 6). Two studies (sample sizes *n* = 88 and *n* = 111) reported that none of the infants born by cesarean (c-) section had hemorrhages (4, 5), while another (*n* = 101) reported a relatively high prevalence in the c-section group (18%) (3). One study indicated increased birth weight and prolonged delivery to be additional risk factors for intracranial hemorrhages (3).

Incidental findings in MR images seldom have major clinical significance, but as they are common (16), they also likely occur in brain MRI research performed in asymptomatic infant populations, and preparing for their occurrence would benefit from better identifying the risk factors. Further, hearing about these findings may be stressful for the parents (17), which underlines the importance of better understanding both the commonness as well as possible implications of these phenomena.

To these ends, we measured the prevalence of incidental brain imaging findings in healthy infants, characterized the findings e.g., according to their location, evaluated their association with obstetric factors, and assessed their possible effects on neurological development during infancy.

## Methods

This study was reviewed and approved by the Ethics Committee of the Hospital District of Southwest Finland and performed in accord with the Declaration of Helsinki.

### Participants

This study was executed as a part of the FinnBrain Birth Cohort Study [www.finnbrain.fi; (18)]. Pregnant women attending their first trimester ultrasound at gestational week (gwk) 12 were recruited at five sites in South-Western Finland. In total, 189 mothers and their infants were recruited to an MRI visit, of which 180 (95.2%) imaging sessions were successfully completed and provided structural MR images, and 175 were included in the subsequent analyses (the rest were excluded due to motion artifacts). The participant families were recruited by a phone call to the mother, at earliest 1–2 weeks after childbirth. The parents gave written informed consent also on behalf of their infant.

The exclusion criteria for the mothers were alcohol or drug abuse; severe psychiatric disorders; medication for psychosis, epilepsy or bipolar disorder. The exclusion criteria for infants included occurrence of any perinatal complications with neurological consequences (e.g., hypoxia), scoring <5 in the 5 min Apgar score; previously diagnosed central nervous system anomaly, an abnormal finding in any previous MRI scan or birth weight <1,500 g. The participating infants were full-term (born between 37 and 43 gwks; with two exceptions born on 36 gwk and two with the information not available, none of which belonged to the incidental finding group). All were born from singleton pregnancies.

Obstetric data were gathered from the Finnish Medical Birth Register of the National Institute for Health and Welfare (www.thl.fi), and included information on duration and mode of delivery, gestational age, use of anesthetics or oxytocin induction during delivery, mother's parity, possible episiotomy, gestational age at birth, child Apgar scores (1 min and 5 min), head circumference, birth weight, birth height and pH of the umbilical blood, among other data that were filled in by the medical staff following delivery at the hospital. Mode of delivery was categorized into (a) vaginal, (b) assisted deliveries (vacuum-assistance), and (c) c-sections (elective or emergency). With anesthetics, two categories were formed: (1) epidural and spinal anesthesia and their combinations and (2) all other anesthetic forms or no pain alleviation. Episiotomy is not a routinely performed procedure during delivery in Finland, as it is in some countries. It was included as one of the obstetric variables as it was considered to be an indirect indicator of increased pressure on the fetus during the second stage of labor.

### Image Acquisition

The infants underwent MRI scans in a Siemens Magnetom Verio 3T scanner (Siemens Medical Solutions, Erlangen, Germany) at age 2–5 weeks, calculated from the due date, at the Turku University Hospital. Upon arrival, an experienced radiographer went through the study protocol with the parents and confirmed the lack of contraindications for the scan. Before the scan, the infants were fed with (breast) milk to help them to sleep and then swaddled into a vacuum mattress to reduce possible limb movement. The scan was performed without anesthetics, and the children were provided with sufficient hearing protection (infant ear wax and custom-sized ear muffs) of ~42 dB noise reduction. The duration of the whole scanning protocol was ~40 min (maximum duration 60 min). The family was free to discontinue the study at any point and the scan was aborted if the baby was not soundly asleep and/or still in the scanner.

Axial PD-T2-weighted sequence with 1.0 × 1.0 × 1.0 mm isotropic voxels, TR of 12,070 ms and effective TE times of 13 and 102 ms were used to produce both PD-weighted and T2-weighted images from the same acquisition. The total number of slices was 128. A sagittal 3D T1 sequence with 1.0 × 1.0 × 1.0 mm voxel size (TR 1,900 ms, TE 3.26 ms, TI 900 ms) was also acquired. Additionally, the imaging included field mapping, a set of DTI images and functional imaging for some participants (data not reported here) (19).

All the successful (*n* = 175) brain structural T1 and/or T2 images were evaluated by a radiologist specialized in pediatric neuroradiology (author RP). When incidental findings were detected, the researchers informed the families about the finding(s) in 1–4 weeks' time. All the families with a finding were offered a child neurological examination and consultation by an experienced pediatric neurologist (author TL), and 11 out of 13 families used this opportunity.

### Neurological Assessment

The pediatric neurologist's visit consisted of a thorough clinical history and interview of the child's health, developmental milestones and possible abnormal symptoms. A complete somatic examination was accompanied by a neurological examination by an experienced pediatric neurologist using a standardized proforma of the Dubowitz neurologic examination for children below 6 months (20) and the Hammersmith Infant Neurological Examination (HINE) for all children (21). The results of the clinical examination together with the brain MRI findings were discussed with the parents who were given an opportunity to contact the consultant also after the visit.

### Statistical Analysis

Statistical analyses were performed with SPSS version 23 (Armonk, N.Y., USA). Descriptive statistics, group comparison analysis and odds ratios for putative risk factors for incidental intracranial hemorrhages were calculated. Statistical significance in the comparisons for the categorical variables was determined with Chi-square test, and for continuous variables, with two-sample *t*-test (means and SDs) or nonparametric Wilcoxon rank-sum test (medians and median absolute deviation (MAD), scaled by a factor *k* = 1.4826) depending on the normality of the distribution of the data. Multiple comparison corrections were not performed due to small sample size. In the risk factor analysis, statistical significance was calculated by using Boschloo's test (22, 23).

## Results

### Demographics

All 175 infants were born from singleton pregnancies without any significant prenatal complications as per inclusion criteria. 94 (54%) were males and 81 (46%) females. The mean (SD) maternal age was 30 (4.2) years. Out of the whole sample, 125 (71%) infants were born through non-assisted vaginal delivery, 21 (12%) with instrumentally assisted delivery using vacuum extraction and 29 (18%) with c-section. Epidural or spinal anesthesia was used as analgesia in 95 (59%) deliveries. In all, 72 (41%) of the mothers were primigravida. The study sample was considered to be representative of the Finnish population, as the proportions of different delivery methods as well as other obstetric factors corresponded well with the national statistics on deliveries (24). The demographic data is presented in Table 1.

**Table 1:** Characteristics of the study subgroups.

	**Incidental findings** ***n* (%) = 13 (7.4)**	**Hemorrhage** ***n* (%) = 12 (6.9)**	**SDH** ***n* (%) = 10 (5.7)**	**IPH** ***n* (%) = 4 (2.3)**	**No findings** ***n* (%) = 162 (93)**
**GENDER**					
Male (%)	6 (46)	6 (50)	5 (50)	1 (25)	88 (54)
Female (%)	7 (54)	6 (50)	5 (50)	3 (75)	74 (46)
Birth weight (g) [mean (SD)]	3427 (476)	3448 (490)	3465 (370)	3446 (689)	3531 (432)
Birth height (cm) [mean (SD)]	50.0 (2.1)	50.2 (2.2)	50.8 (1.0)	49.5 (3.8)	50.5 (1.8)
Head circumference (cm) [median (MAD)]	34.8 (1.3)	34.9 (1.2)	34.9 (1.3)	34.8 (0.6)	35.1 (1.3)
Age at imaging [median (MAD)] from due date (days)	21.0 (4.4)* p = 0.014	21.5 (4.4)* p = 0.042	22.0 (3.0)	19.5 (7.4)	25.0 (7.4)
From date of birth (days)	23.0 (5.9)* p = 0.025	22.0 (5.9)* p = 0.019	22.0 (5.9)* p = 0.043	23.5 (2.2)	26.0 (7.4)
**LENGTH OF PREGNANCY**					
Weeks [median (MAD)]	40.0 (0.0)	40.0 (0.0)	40.0 (0.0)	43.5 (0.7)	39.5 (0.7)
Days [mean (SD)]	281 (4.4)	282 (4.4)	282 (3.7)	282 (5.9)	280 (8.2)
**MODE OF DELIVERY**					
Spontaneous vaginal delivery (%)	9 (69.2)	8 (66.7)	6 (60)	4 (100)	116 (71.6)
Assisted delivery (vacuum) (%)	4 (30.8)* p = 0.04	4 (33.3)* p = 0.029	4 (40)* p = 0.012	0	17 (10.5)
Section (%)	0	0	0	0	29 (17.9)
**LENGTH OF DELIVERY**					
Total (min) [median (MAD)]	462 (312)	458 (303)	418 (266.9)	466 (310)	448 (288)
Cervical dilation (min) [median (MAD)]	416 (285)	400 (245)	383 (252)	435 (274)	415 (274)
Active labor (min) [median (MAD)]	31.0 (17.8)	31.0 (22.2)	34.0 (26.7)	27.0 (13.3)	23.0 (23.7)
Primigravida (%) [median (MAD)]	6 (46.2)	6 (50.0)	6 (60.0)	0 (0.0)	66 (40.7)
Apgar score 1 min [median (MAD)]	9.0 (0.0)	9.0 (0.0)	9.0 (0.0)	9.0 (0.0)	9.0 (0.0)
Apgar score 5 min [median (MAD)]	9.0 (0.0)	9.0 (0.0)	9.0 (0.0)	9.0 (0.0)	9.0 (0.0)
Artery pH [mean (SD)]	7.2 (0.1)	7.2 (0.1)	7.2 (0.1)	7.3 (0.1)	7.3 (0.1)
**ANESTHETICS**					
Epidural/spinal (%)	8 (61.5)	7 (58.3)	5 (50.0)	3 (75.0)	95 (58.6)
No anesthetics/mild anesthetics (%)	4 (30.8)	4 (33.3)	4 (40.0)	0	65 (40.1)
NA	1 (7.7)	1 (8.3)	1 (10.0)	1 (25.0)	2 (1.2)
Oxytocin induction (%)	5 (41.7)	4 (36.4)	2 (22.2)	2 (66.7)	60 (37.5)
NA	1	1	1	1	2

SDH or IPH, Hemorrhage; SDH, subdural hemorrhage; IPH, intraparenchymal hemorrhage; SD, standard deviation; MAD, mean absolute deviation (scaled by a factor k = 1.4826); NA, not available. Statistically significant differences between groups of no findings and groups with the indicated finding (all incidental findings, hemorrhages, subdural hemorrhages, and intraparenchymal hemorrhages) are marked with *.

### The Prevalence of Incidental Findings

Incidental brain imaging findings were detected in 13 (6 male, 7 female) out of 175 infants, the prevalence of all findings thus being 7.4%, and hemorrhages in 12 infants, making the prevalence of hemorrhages 6.9%. All deliveries within the group of incidental findings were vaginal, of which four were assisted with vacuum [the prevalence in vacuum-assisted deliveries 19% (4/21)].

### Description of the Incidental Findings

**Figure 1:**
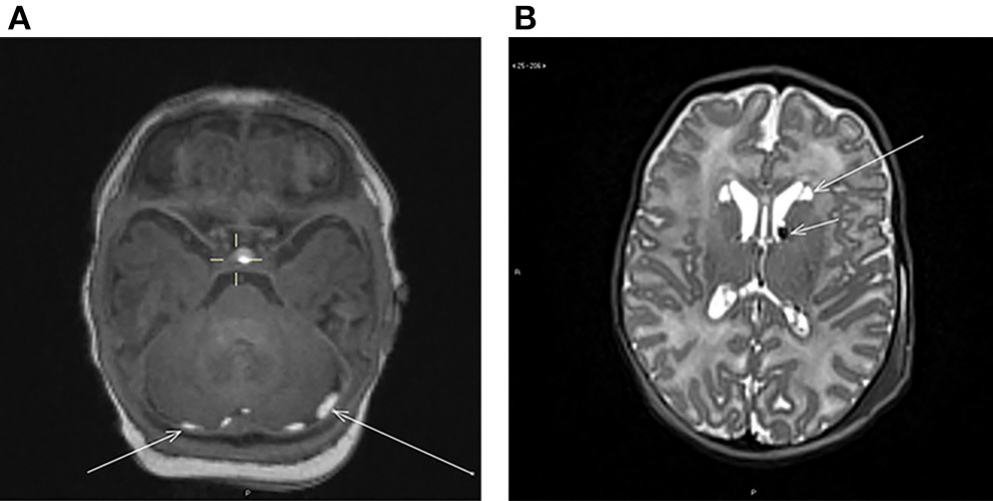
**(A)** Axial T1-weighted image showing bilateral subdural hemorrhages (arrows) in the posterior fossa behind the cerebellum. **(B)** Axial T2-weighted image showing bilateral cysts at the head of nucleus caudatus (long arrow) and bilateral hemorrhages at the caudothalamic groove (short arrow).

The findings included subdural and intraparenchymal hemorrhages, cysts and their co-existence. Out of all the incidental findings, subdural hemorrhages had the highest prevalence (*n* = 10, 5.7%). Subdural hemorrhages were mostly located in multiple sites (Table 2). The majority (*n* = 10) was located in the posterior fossa (Figure 1), but also temporal (*n* = 4) and occipital (*n* = 3) hemorrhages were detected. The temporal subdural hemorrhages coincided mainly with hemorrhages located in the posterior fossa and in the occipital lobe (3 out of 4). Two infants with subdural hemorrhages had also additional parenchymal involvement; one cyst-associated hemorrhage in the caudo-thalamic region (Figure 1) and one hemorrhage in the cerebellar region, respectively. Furthermore, one independent cerebellar parenchymal hemorrhage, one caudo-thalamic cyst and one caudo-thalamic cyst combined with a parenchymal hemorrhage were observed. The one infant with an independent caudo-thalamic cyst was excluded from the subsequent analyses due to the high prevalence of these cysts in normal brain development (7, 8), and as birth-related factors are likely uninvolved in their occurrence. The distribution of the hemorrhages is described in Table 2 and examples of a posterior fossa hemorrhage and a cyst associated with hemorrhage are given in Figure 1.

**Table 2:** Locations of the hemorrhages.

**Participant**	**SDH**			**IPH**	
	**Posterior fossa**	**Occipital**	**Temporal**	**Caudo-thalamic**	**Posterior fossa**
1	×	×	×		
2	×	×		×	
3	×	×	×		
4					×
5			×		
6				×	
7	×				
8	×				×
9	×				
10	×		×		
11	×				
12	×				
Total	9	3	4	2	2

*SDH, subdural hemorrhage; IPH, intraparenchymal hemorrhage*.

### Risk Factors for Hemorrhages

Among the evaluated parameters, vacuum-assisted delivery was found to be a risk factor for subdural hemorrhages with an odds ratio (OR) of 4.7 (95% CI [1.18;18.9], *p* = 0.032) and especially for temporally located hemorrhages (OR = 20.7, 95% CI [2.2;378], *p* = 0.0078) (Table 3). Of altogether four temporal subdural hemorrhages, three (75%) occurred with vacuum-assisted delivery, while the posterior fossa hemorrhages occurred mainly in spontaneous vaginal deliveries. All temporal hemorrhages were observed in infants with primiparous mothers who all had also undergone episiotomy.

**Table 3:** Risk of hemorrhages in relation to obstetric variables given as odds ratios (OR, [95% CI]).

	**Hemorrhage**	**SDH**	**Temporal SDH**
Vacuum-assistance	3.4 [0.92;11.7] *p* = 0.059	4.7 [1.18;18.9]* p = 0.032	20.7 [2.2;378]* p = 0.0078
Primigravidity	1.47 [0.44;4.9] NS	2.3 [0.57;8.5] NS	
Epidural/spinal anesthetics	1.18 [0.34;4.2] NS	0.83 [0.23;3.3] NS	0.66 [0.08;5.7] NS

Statistically significant differences marked with *. SDH, subdural hemorrhage; NS, non-significant. Statistical significance is calculated with Boschloo's test. Vacuum-assistance is compared to non-assisted vaginal deliveries, primigravidity, and anesthetics are assessed in the whole dataset.

No imaging findings were detected in the section-delivered infant group. Birth weight, height, infant sex, duration of gestation or duration of delivery, maternal age, used anesthetics, infant Apgar score at 1 or 5 min, use of oxytocin induction or maternal parity were not related to the occurrence of hemorrhages. The median (MAD) infant age at imaging calculated from the date of birth in the hemorrhage group [23.0 (5.9) days] was slightly lower than in the group with no incidental hemorrhages [26.0 (7.4) days]. Age was not controlled in the risk factor calculations due to small group sizes, but no significant differences were observed in ages between the groups in risk factor analysis.

### Condition of the Infants With Incidental Findings

All infants with an incidental finding had been discharged from the maternity ward according to a normal procedure, without any observed long-lasting symptoms. Infants stayed in the maternity hospital, on average, 3.1 days (range 0;6). One newborn, who initially required acute surveillance due to breathing difficulties, was posteriorly diagnosed with cerebellum intraparenchymal hemorrhage. His clinical presentation at birth was deemed to be caused by an infection and he was evaluated as normal upon a neurological consultation visit.

### Follow-Up on Neurological Development

None of the 11 infants who attended the pediatric neurologist's visit had any clinically identifiable, significant neurological symptoms or deficits in their development at the time of the examination (infant age range 7–54 weeks, mean 16.6 weeks). Infant age at neurological examination varied due to scheduling issues but the comparability of the data on neurological development was ensured by using two different, age-appropriate assessment protocols. One family choosing not to book a visit was contacted by phone by the pediatric neurologist. The family explained they did not regard the visit necessary as the child was considered to develop normally in the controls of well-baby clinics.

The general health of all examined children was normal. The neurological assessment of children below 6 months of age (*n* = 9) was performed by the Dubowitz neurologic examination proforma (20). None of the children had clinically significant deviation in the scoring. Four children had one deviant item (mainly mild truncal hypotony) and five children had no deviant items. It has been previously reported that 1–2 deviant items are found in one third of the normal population examined according to Dubowitz infant neurological examination proforma (20). In the Hammersmith Infant Neurological Examination (HINE) (21), cranial nerve function was normal in all children, and the motor milestones were fully normal in 10 and mildly delayed in one child, who also was the only one presenting mild problems with social behavior. The neurological assessment of posture, movement, tone and reflexes were performed and the median optimality HINE score was 65 (range 0.94), while the previously reported optimality score varies between 61 and 74 in normally developing children between 3 and 8 months. No marked delay in developmental milestones or in behavior was detected in the studied children.

Of note, all infants, including the two whose parents declined the pediatric neurologist's visit, were also followed up in Finnish well-baby clinics that perform multiple check-ups during the first year of life.

## Discussion

We found that 6.9% of our neonate participants had intracranial hemorrhages as incidental findings in 3 T MRI measurements. None of the hemorrhages proved to be of clinical significance in the follow-up, which supports the hypothesis that they are not a major concern for current delivery treatment guidelines.

Our study reports a lower occurrence of intracranial hemorrhages compared to previous studies, where the prevalence has ranged between 8.1 and 46% (3–6), although the prevalence of 6.9% in our sample is still noteworthy (Table 4). The variation in estimates between studies could be partly explained by differences in infant age at time of scanning, as the highest prevalence (46%) was detected when imaging was conducted during the first 72 h postpartum (3). In that same study, all findings were subdural hemorrhages (*n* = 46) (3), while we detected also four intraparenchymal hemorrhages. The former study used 1.5 T magnetic field strength whereas 3 T was employed in ours, partially explaining the detection of intraparenchymal hemorrhages; increasing magnetic field strength improves resolution and facilitates the detection of more subtle incidental findings (3–5). As our scanning time points were later (mainly on weeks 3 and 4 after birth), the discrepancies in prevalence estimates could partly stem from hemorrhage resorption over the first days and weeks of life. In our sample, the age at imaging was lower in the hemorrhage group than in the control group, supporting this notion. Yet, Looney et al. estimated the prevalence of intracranial hemorrhages to be 26% with a magnetic-field strength and imaging time period both close to this study. One explanatory factor for this discrepancy might lie in the thorough prenatal assessments that are routine in Finland, e.g., selecting c-section candidates with care.

**Table 4:** Comparison between studies reporting neonatal intracranial incidental findings.

**Publication**	**Magnetic field**	**Study population**	**Age at imaging**	**Prevalence of incidental findings**	**Incidental finding types**	**Associated risk factors**	**MRI indications**
Our study	3.0 T	175	2–5 weeks	7.4% (13/175)	10 SDH 4 parenchymal 3 cysts	vaginal delivery, vacuum assistance	None, cohort study of healthy population
Looney et al. (4)	3.0 T	88	1–5 weeks	26% (17/88) (vaginal deliveries only)	16 SDH 1 germinal matrix 2 SAH 5 intraparenchymal (co-existing)	Vaginal birth	None, prospective study of brain development
Rooks et al. (3)	1.5 T	101	72 h	46% (46/101)	All SDH	Vaginal birth	None, healthy asymptomatic infants
Whitby et al. (5)	0.2 T	111	48 h	8.1% (9/111)	All SDH	Vaginal birth, forceps after failed attempt with vacuum	None, healthy asymptomatic infants
Sirgiovanni et al. (6)	1.5 T	240 (152 term, 88 preterm)	1–40 days (mean 10 days)	15% (36/240)	All SDH + 4 co-existing intraparenchymal	Vaginal birth, older gestational age, bigger weight	Neurological symptoms, perinatal asphyxia, abnormal US finding, metabolic symptom, infection
Tavani et al. (9)	1.5 T	24	1–22 days	62% (13/21) (vaginal deliveries only)	17 SDH 1 intraparenchymal 1 intraventricular 7 choroid plexus	Vaginal birth	Known congenital heart disease
Malova et al. (25)	1.5 T	276 (preterm, very low birth weight infants)	–	10% (28/276)	3 developmental venous anomalies 4 arachnoid cysts 6 pituitary abnormalities 15 others	–	Very low birth weight infants in neonatal intensive care unit
Wintermark et al. (26)	3 T	12	1–2 days	17% (2/12)	1 hemorrhage-combination (epidural, subdural, intraparenchymal) 1 sinus thrombosis	–	Asphyxia

*SDH, subdural hemorrhage; SAH, subarachnoidal hemorrhage*.

Vaginal delivery and vacuum-assistance were the only significant risk factors for intracranial hemorrhages, from a number of obstetric variables evaluated in our study. In Finland, the rate of c-sections almost doubled between the years 1998 and 2005, from 6.8 to 11.3% (27), with the latest rate of 15.9% for sections (6.7% for emergency sections) (24), and a similar trend in the popularity of c-sections can be seen worldwide (28). The current reasoning behind selecting c-section as the delivery method more frequently is to ensure the safety of the neonate. However, this has not lead to improvements in neonatal outcomes in the short term, but instead has increased admissions to neonatal intensive care units (NICUs) (27). Cesarean sections are known to predispose neonates to respiratory distress and tachypnea (29). Therefore, it is not warranted to opt for c-section over vaginal delivery based on risk of intracranial hemorrhages.

When instrumental assistance is required during delivery, vacuum-assistance is currently the preferred method. The most common indications for the use of vacuum are problems with recording fetal heart rate and prolonged second stage of labor, and vacuum assistance is associated with primiparity, regional analgesia, smaller birth weight, and induction of labor acting as contributory factors (30, 31). However, again, the potential occurrence of intracranial hemorrhages is not an adequate indication for modifying delivery practices, as they are not shown to compromise infant health. Further, prolonged labor may be the more important common denominator here. The role of the actual instrument assistance remains elusive, as previous studies have provided discordant estimates on whether assisted delivery increases the risk for subdural hemorrhages in the first place, or if spontaneous vaginal delivery is the strongest determinant (3–6). In this study, vacuum-assistance of delivery increased the risk for hemorrhages, although the hemorrhages were clearly associated with all vaginal deliveries when compared to c-sections, which is line with prior studies (3–6, 9), albeit exceptions exist (32).

Many limitations in extant studies weaken the comparison of consequences between different delivery modes, including incomplete reporting of events during labor that may substantially contribute to the choice of delivery method. Future prospective studies are needed to address this issue.

The neurological assessment of all children was normal measured by 1–2 standardized systems (20, 21). However, the age for the clinical examination (average 3–4 months) is not generally the most ideal for prognostic evaluation of infants. Nevertheless, it was considered as ethically most sound to invite the families as soon as possible in terms of the study protocol. Therefore, two different assessment methods for two different age groups were used to detect any possible deviant signs or symptoms in neurological development. Altogether, neither marked delay in developmental milestones nor abnormal behavior was detected in the studied children. Supporting earlier notions, our study suggests that the clinical consequences of these findings may be marginal (33).

Incidental findings are becoming continuously more frequent as the usage and accuracy of MRI increase. This is especially relevant in research scans using high resolution MRI sequences as a common practice, as the prevalence of incidental findings is higher in them compared to when MRI is performed as a part of clinical screenings, with lower resolution (4.7 and 1.7%, respectively) (34). Current challenges regarding the incidental findings include scarce information on their consequences on the individual level, and lack of best practices on both sharing the information with the participants as well as in guaranteeing that the study participants understand the possible repercussions of participation beforehand (35).

We followed a recommended protocol in the handling of incidental findings (36), including consulting a specialist and discussing the findings with multiple researchers. One suggested way of handling incidental findings is to evaluate the net benefit received by the participant and thus not to disclose the findings with unlike net benefit (36). However, we found that it was a participant's right to become aware of the findings, if willingness had been shown to receive this kind of information, when inquired in advance. Still, when giving people information they did not particularly ask for, the message needs to be accurate, and enough support and guidance should be given. In the future, when proceeding to the MR imaging of older children within the FinnBrain Birth cohort study, the correct handling of the incidental findings will be even more important, as incidental findings may include potential malignancies, etc. (37).

Parental distress can have grave effects on parent-child bonding and attachment behavior (38, 39), which emphasizes the importance of providing parents with knowledge about the clinical relevance of the findings. In this study, the opportunity to discuss the incidental MRI findings with an expert clinician was offered to all families. As no family contacted the clinic for more information after the consultation visit, this approach was regarded as sufficient to reduce anxiety in parents. In general, after receiving normal results from the neurological examination, sufficient effort and resources should be placed on informing parents about the positive prognosis of the child. Furthermore, all feelings of self-accusation and failure should be avoided, and the fact that hemorrhages are quite common in completely normal deliveries without any complications should be highlighted.

Our study design provided an opportunity to evaluate asymptomatic intracranial findings in infants within a representative sample of the Finnish term-born population and likely provided an accurate estimate of the prevalence without deviation due to selecting individuals based on symptoms. Finland, among other Nordic countries, has the lowest neonatal mortality rate (28) and qualitative differences between countries in peripartum care may contribute to differences also seen in the occurrence of hemorrhages (although this aspect was not in the scope of the current study). The sample size is acknowledged to be modest for epidemiological evaluations and does not encompass the age range immediately after birth nor does it include repeated measurements. Further, the neurological follow-up examination of the infants with incidental findings was performed only once and at a relatively young age, so that future developmental problems could not be excluded. The inclusion of additional risk groups such as infants with peripartum complications or even subgroups of healthy neonates with excess crying (maybe due to pain) would be beneficial for future studies. The relatively late scanning time point is also a limitation of this study. Our imaging scheme does not include SWI (susceptibility weighted imaging) or T2^*^ weighted sequences which would improve the detection of intraparenchymal microhemorrhages. SWI sequence does not show hypoxia, which is a more direct sign of brain damage, and which on the other hand is shown in T1 weighted images in addition to subdural hemorrhages (40). Thus, we regarded T1 weighted sequence to serve our purposes better in detecting major intracranial changes as the imaging time was limited. Magnetic resonance imaging was performed at ca. Three weeks postpartum, when delivery-associated hemorrhage volumes may be diminished due to resorption (3, 5). In two previous studies, the scanning was performed within 48–72 h from birth, with the finding percentage ranging between 8.1 and 46% (3, 5), while one study has quite a similar age period to ours and incidental findings in 26% of infants (4). The limitations of our study appear to correspond with those of the previous studies. Thus, in the future, a prospective research setting considering these modifying or confounding factors beforehand is needed to acquire more specific data concerning incidental brain findings related to birth. Future studies could address how fast incidental hemorrhages disappear after birth, to further delineate, e.g., the apt timing for follow-up scans. Also, the effect of variation in blood coagulation on the appearance of incidental findings could be addressed in future. Regarding possible relevance of location or size of incidental intracranial hemorrhage on child outcomes, larger study populations are required.

## Conclusion

Minor intracranial hemorrhages are detected frequently in infant MR scans. Vaginal delivery and vacuum assistance seem to increase the prevalence of hemorrhages. Most hemorrhages are benign with little clinical significance within the first years of life and have unlikely consequences for later neurodevelopment either. Investigators should take into account the possibility of detecting such hemorrhages and plan how to address them with apt expertise and careful communication with the families.

## Data Availability Statement

The datasets generated for this study will not be made publicly available because of restriction imposed by the Finnish law and the study's ethical permissions do not allow sharing of the data used in this study. Requests to access the datasets should be directed to the Principal investigator of the FinnBrain Birth Cohort Study HK (hasse.karlsson@utu.fi).

## Ethics Statement

The studies involving human participants were reviewed and approved by the Ethics Committee of the Hospital District of Southwest Finland. Written informed consent to participate in this study was provided by the participants' legal guardian/next of kin.

## Author Contributions

HK and LK devised the project and main conceptual ideas. HK, LK, JT, and NS designed the infant imaging study setting. SL, JT, and NS collected the imaging data. VK, SL, JT, and NS contributed to the analysis of the data. VK, SL, AC, ES, JT, NS, TL, and RK participated to writing the manuscript. TL performed the neurological examinations. RP reviewed the MR images and evaluated the incidental findings. JS, HM, and JT designed the imaging scheme, provided the technical details, and contributed to the analysis tools. HK, NS, and JT supervised the project. All authors provided critical feedback, helped shape the manuscript, and accepted it in it's final form.

### Conflict of Interest

The authors declare that the research was conducted in the absence of any commercial or financial relationships that could be construed as a potential conflict of interest.
